# College students’ learning stress, psychological resilience and learning burnout: status quo and coping strategies

**DOI:** 10.1186/s12888-023-04783-z

**Published:** 2023-06-02

**Authors:** Zhen Gong, Huadi Wang, Mingxia Zhong, Yuling Shao

**Affiliations:** 1 College of E-commerce, Zhejiang Business College, Hangzhou, 310053 Zhejiang China; 2 Center for Rehabilitation Medicine, Rehabilitation & Sports Medicine Research Institute of Zhejiang Province, Department of Rehabilitation Medicine, Zhejiang Provincial People’s Hospital, Affiliated People’s Hospital, Hangzhou Medical College,, Hangzhou, 310024 Zhejiang China; 3 No. 158 Shangtang road, Gongshu district, Hangzhou, Zhejiang Province China

**Keywords:** College, Student, Stress, Psychological resilience, Burnout, Care, Management

## Abstract

**Background:**

The relationships of college students’ learning stress, psychological resilience and learning burnout remain unclear. We aimed to investigate the status quo and relationship of college students’ learning stress, psychological resilience and learning burnout, to provide insights to the management and nursing care of college students.

**Methods:**

From September 1 to October 31, 2022, students in our college were selected by stratified cluster sampling and underwent survey with the learning stress scale, college students’ learning burnout scale and the psychological resilience scale of college students.

**Results:**

A total of 1680 college students were surveyed in this study. The score of learning burnout was positively correlated with the score of learning stress (r = 0.69), and negatively correlated with the score of psychological resilience (r = 0.59), and the score of learning stress was negatively correlated with the score of psychological resilience (r = 0.61). Learning pressure was correlated with the age(r=-0.60) and monthly family income(r=-0.56), the burnout was correlated with the monthly family income(r=-0.61), and psychological resilience was correlated with the age(r = 0.66) (all P < 0.05). Psychological resilience played an intermediary role in the prediction of learning burnout by learning stress, with an total intermediary role of-0.48, accounting for 75.94% of the total effect.

**Conclusions:**

Psychological resilience is the mediating variable of the influence of learning stress on learning burnout. College managers should take various effective measures to improve college students’ psychological resilience to reduce college students’ learning burnout.

## Introduction

With the increasingly fierce social competition and the gradual popularization of higher education, college students will face certain learning pressure, including learning pressure from their own internal and external environment [1]. Under the long-term learning pressure, students show withdrawal behavior or unwillingness to invest time and energy in learning, resulting in emotional, attitude, physical and other aspects of exhaustion, and gradually lose their learning goals and ideals [2]. Psychological resilience is a personal character and a positive quality, which to a large extent determines how to face challenges and deal with stress in different environment and conditions. Many studies [3–5] have shown that individuals with a high level of psychological resilience have a more positive cognitive style of reality and the future, and their level of self-esteem is correspondingly higher, they are more inclined to flexibly use coping strategies directed by problems and emotions, to make more effective use of a variety of resources to overcome difficulties, reduce psychological distress and improve adaptability. Some studies [6, 7] have found that there is a significant negative correlation between mental resilience and mental health, and psychological resilience has a high predictive effect on mental health.

At present, the researches on learning stress and learning burnout are mainly to explore the role of variables that affect the relationship between learning stress and learning burnout. When some scholars [8] studied the factors affecting college students’ burnout, they have found that social support can predict the burnout of college students. In addition, some scholars [9] have put forward six factors that affect students’ learning burnout, among which learning load is one of the more important. The results of the survey show that students who go through a variety of qualification examinations will feel great learning pressure, resulting in learning burnout, and learning burnout will become a negative factor affecting their academic performance. Whether it indicates that college students’ psychological resilience is also an intermediary variable between learning stress and learning burnout needs further investigations. Therefore, this study aimed to explore the relationship among learning stress, psychological resilience and learning burnout of college students, to provide valuable reference for psychological management and nursing care of college students.

## Methods

This study design was a cross-sectional survey, the study was performed according to the related regulation and guidelines. The study has been reviewed and approved by the ethics committee of Zhejiang Business College (approval number: 2022zsy-kj-08). And written informed consents had been obtained from all the included college students. In the course of the survey, the surveyed students had the right to refuse to participate in the survey, we strictly followed the principle of voluntary participation and informed consent. All materials were kept secret after collection and were used only for academic research.

### Study population

In this study, from September 1 to October 31, 2022, students in our college were selected by stratified cluster sampling. In this study, the subjects were selected strictly according to the inclusion criteria. The inclusion criteria of the study population were as follows: (1) students must be full-time college students; (2) they were currently studying normally in our college; (3) the students have no history of mental illness; and (4) they volunteered to participate in this study. We evaluated the college students’ learning stress, psychological resilience and learning burnout status quo with questionnaires.

### Sample size calculation

This study adopted the method of stratified cluster sampling to calculate the sample size [10]. We initially selected a total of about 1600 freshmen to seniors of various disciplines in our college. Considering that about 6% of the questionnaires were lost and invalid, at least 1696 copies of questionnaires should be actually distributed.

### Survey tools

The survey tools of this study mainly included four parts: General data questionnaire; College students’ learning stress scale; college students’ psychological resilience scale; college students’ learning burnout scale.

Referring to many domestic and foreign literature, consulting psychology and nursing experts, and combining with the purpose of this study, the researchers designed a general information questionnaire for medical students, including medical students’ age, gender, body mass index (BMI), whether the student was the only child of family, parents’ educational level and monthly family income.

The learning stress scale for college students was compiled by Lan Tian etc. [11], including seven subscales: Pressure of learning prospects, pressure of academic competition, pressure of learning effectiveness, pressure of learning atmosphere, pressure of schoolwork, pressure of learning condition, pressure of family expectation. Five points were used to score 1–5 points from “completely non- compliance” to “complete compliance”. The higher the score, the greater the learning pressure. The construct validity of the questionnaire was good (comparative fit index (CFI) = 0. 99), and the internal consistency coefficient of the scale was 0.93.

The college students’ learning burnout scale was compiled by Lian Rong et al [12]. The scale had a total of 20 questions, which were divided into three dimensions: depression, misbehavior and low sense of achievement. It used five points to score 1–5 points from “completely inconsistent” to “completely consistent”. The higher the score, the more burnout. The construct validity of the questionnaire was good (CFI = 0.96), and the internal consistency coefficient was 0.75.

The scale of psychological resilience of college students was compiled by Hu Yueqin et al [13], which was based on the process model of psychological resilience. The scale was composed of 27 questions and divided into 2 factors and 5 factors of individual manpower and support. Individual manpower included three factors: goal focus, emotional control and positive cognition, and support includes two factors: family support and interpersonal assistance. The five factors reflected the effectiveness of cognition, emotion, behavior and environment of adolescents in adversity to help them achieve good adaptability and resist adversity. The scale was scored with 5 points, 1 for “completely inconsistent”, 2 for “relatively inconsistent”, 3 for “inexplicable”, 4 for “more consistent” and 5 for “complete compliance”. The construct validity of the psychological resilience scale was good (CFI = 0.93), and the internal consistency coefficient was 0.70.

On the basis of obtaining the cooperation of the students, the questionnaire was distributed, the investigators were trained by the researchers themselves, the unified instructions were used, and the questionnaires were distributed on the spot and uniformly collected. In the process of data collection, the researchers explained the confidentiality of this survey to eliminate the concerns of the subjects. Using unified and standardized instructions, the questionnaire was distributed and collected on the spot, and the omissions were supplemented in time. After the recovery of the questionnaire, the researchers re-checked the integrity and logic of the questionnaire, removed the invalid questionnaire and coded the data to ensure the accuracy of the input and data processing.

### Statistical analysis

The data of this study were analyzed by SPSS22.0 statistical software. The data conforming to normal distribution were expressed by mean ± standard deviation. t test and Chi-square test were used to compare the differences groups, and Pearson correlation analysis was used to evaluate the correlation between college students’ learning stress and psychological resilience and learning burnout. Hayes’ Process Macro for SPSS been used to investigate the mediating role of psychological resilience in the relationship between learning stress and learning burnout. The Bootstrap method was used to test the potential mediating effect. In this study, there was statistical significance when P < 0.05.

## Results

A total of 1696 copies of questionnaires were distributed and collected, Among them, 1680 questionnaires were valid. As shown in Table 1, among the 1680 college students surveyed in this study, female students accounted for 56.67%. Most of the college students were 20–25 years old, the BMI of students was mostly in the normal range, most of the students were the only children of family, their parents’ education level was mainly senior high school, and the family’s monthly income was 5000 ~ 10,000 RMB.

**Table 1:** The characteristics of college students(n = 1680)

Characteristics	Cases	Percentage (%)
Gender		
Female	952	56.67%
Male	728	43.33%
Age(y)		
< 20	396	23.57%
20 ~ 25	1024	60.95%
> 25	260	15.48%
BMI(kg/m_2_)		
< 20	272	16.19%
20 ~ 25	1185	79.54%
>25	223	13.27%
Only child of family		
Yes	1061	63.15%
No	619	36.85%
Parents’ educational level		
Primary school	118	7.02%
Junior high school	469	27.97%
Senior high school	830	49.40%
University	263	15.65%
Monthly family income (RMB)		
< 5000	474	28.21%
5000 ~ 10,000	909	54.11%
> 10,000	297	17.68%

BMI, body mass index

The total score of learning pressure (Table 2) was 129.05 ± 26.61, the total learning burnout scores (Table 3) of college students was 59.41 ± 10.13, the total psychological resilience scores (Table 4) of college students was 90.06 ± 11.85. College students have medium-level learning pressure and learning burnout, and their psychological resilience was generally good.

**Table 2:** The learning pressure scores of college students

Items	Average scores
Pressure of learning prospects	28.53 ± 6.11
Pressure of academic competition	23.07 ± 5.37
Pressure of learning effectiveness	24.76 ± 6.13
Pressure of learning atmosphere	12.12 ± 4.38
Pressure of schoolwork	15.30 ± 6.24
Pressure of learning condition	11.14 ± 5.08
Pressure of family expectation	13.28 ± 4.96
Total score of learning pressure	129.05 ± 26.61

**Table 3:** The learning burnout scores of college students

Items	Average scores
Low learning spirits	23.15 ± 4.86
Improper learning behavior	18.28 ± 4.57
Low sense of learning achievement	16.74 ± 3.99
Total score of learning burnout	59.41 ± 10.13

**Table 4:** The psychological resilience scores of college students

Items	Average scores
Target focus	17.34 ± 3.87
Emotion control	18.01 ± 4.05
Positive cognition	14.78 ± 4.33
Family support	20.68 ± 3.46
Interpersonal assistance	19.29 ± 5.01
Total score of psychological resilience	90.06 ± 11.85

Pearson correlation analysis results showed that the score of learning burnout was positively correlated to the score of learning stress (r = 0.69), and negatively correlated with the score of psychological resilience (r = 0.59), and the score of learning stress was negatively correlated to the score of psychological resilience (r = 0.61). Besides, learning pressure was correlated to the age(r=-0.60) and monthly family income(r=-0.56) in college students, the burnout was correlated to the monthly family income(r=-0.61) in college students, and psychological resilience was correlated to the age(r = 0.66) in college students(all P < 0.05).

As shown in Table 5, psychological resilience played an intermediary role in the prediction of learning burnout by learning stress, with an total intermediary role of -0.48, accounting for 75.94% of the total effect.

**Table 5:** The mediating effect of psychological resilience scores in the relationship between learning pressure and learning burnout of college students

Influencing path	Standardized effect size	Standard error	Percentage of the total effect		95%CI	
					Upper limit	Lower limit
Total effect	-0.63	0.07	-		-0.72	-5.82
Direct effect	-0.28	0.04	24.06%		-0.36	-0.17
Mediating effect	-0.48	0.05	75.94%		-0.60	-0.34

## Discussions

Previous studies [14, 15] on college students have found that college students’ learning pressure is a direct cause of students’ learning burnout, the greater the learning pressure, the more serious students’ learning burnout, and there is a significant negative correlation between learning stress and psychological resilience. Students with high learning pressure show a lower level of psychological resilience [16]. The lower the students’ psychological resilience, the more serious the learning burnout. The results show that there is a significant positive correlation between learning stress and the total score of learning burnout. This shows that with the rise of learning pressure, the level of learning burnout of college students increases accordingly, and the most direct manifestation of learning pressure is that students’ self-confidence is frustrated due to learning pressure, which leads to students’ low learning spirits. Low learning spirits will seriously damage students’ interest in learning and produce emotional experience of learning burnout, resulting in improper behaviors such as learning procrastination.

Learning burnout is significantly negatively correlated with psychological resilience, indicating that the higher the level of psychological resilience of college students, the less the degree of learning burnout, which is mainly manifested in the close relationship between students and teachers and students. During COVID-19 pandemic, some college students are isolated at home, or their academic tasks are mainly online classes, lack of face-to-face communication with teachers and classmates, academic pressure is greater, negative emotions are difficult to dredge, which further aggravates learning pressure. At the same time, the family income of some parents may decrease during COVID-19 pandemic, and the economic pressure from the family may adversely affect the learning psychology of students. The lower the level of psychological resilience, the higher the level of learning burnout, which is mainly manifested in that students are easily alienated from people or things, and their sense of learning self-efficacy is relatively low, which is easy to cause physical and mental exhaustion [17]. Students with low level of psychological resilience hold a pessimistic attitude towards difficulties when they encounter setbacks and difficulties, often fail to adhere to goals, concentrate on solving problems. They may have large mood fluctuations and cannot vent their bad emotions through meaningful interpersonal relationships, so it is easy to produce a sense of learning burnout [18, 19]. On the contrary, students with a higher level of psychological resilience will be full of confidence, maintain a positive attitude when they encounter setbacks and difficult situations, persist and concentrate on solving the problems encountered, and will take the initiative to seek family and other interpersonal relationships to vent their negative emotions [20].

Psychological resilience plays an intermediary role between learning stress and learning burnout. Under the effect of psychological resilience, learning pressure maintains the individual’s way of understanding problems and the normal level of self-esteem, and they tends to use more positive strategies to deal with the negative emotions brought about by learning and solve the problems encountered in learning, to reduce or eliminate individual learning burnout [21]. Previous results [22, 23] show that psychological resilience can directly reduce the symptoms of psychological distress caused by stress, promote individual adaptation to a certain extent, and reduce the negative effects of stress disorders on individual mental health. Individuals with high psychological resilience are more accustomed to explaining events with an optimistic and positive attitude, so they can experience more positive emotions, while individuals with low psychological resilience experience more negative emotions, they are also more sensitive to stressful events in daily life [24–26]. Psychological resilience can provide college students with resources to deal with stress events, and promote college students to make more effective use of various resources to overcome difficulties and recover quickly from learning stress [27–29]. Recent studies [30–32] have also shown that higher scores on the personality traits of novelty seeking and neuroticism can be associated with higher levels of burnout in college students. It seems that considering personality traits in future studies can be useful in improving burnout among college students. Hence, it is necessary to pay more attention to this matter in the future scope of the study.

College managers and teachers should attach importance to the development of college students’ psychological resilience and shape their good psychological quality so that they can gain a foothold in the society [33]. The results of this study have shown that age and family income have an important influence on college students’ learning stress, boredom and psychological resilience. College students should realize the importance of improving psychological resilience to the development of their physical and mental health. Colleges managers and teachers should actively set up courses related to mental health education for college students to guide students to plan their future goals and shape a positive psychological resilience [34]. The college can carry out a variety of learning forms, such as motivational video sharing, extracurricular sitcom performances, group psychological counseling, etc., to point out the direction for the cultivation of college students’ positive mentality [35]. Besides, the college should provide a variety of platforms for college students to exercise themselves, carry out various social practice activities, create a harmonious campus atmosphere, and let college students experience the truth of life in practice [36–38]. Furthermore, college students should cultivate their own positive qualities, establish good interpersonal relationships, and learn various ways to solve problems [39, 40]. If encountered difficulties, individuals should learn to solve the problem-centered way of thinking, do not lose confidence because of setbacks, or give up not discouraged, dare to try and challenge, actively analyze and solve problems. Through carrying out a variety of targeted courses and activities to enhance the learning pressure and burnout of college students, and improve the psychological quality of college students [41, 42].

There are some shortcomings in this study that are worth considering. First of all, this study has conducted a sample survey on the basis of considering the gender and major of the research population, but the sample size is small, and the representativeness of the research population may be insufficient. Secondly, this study mainly used the quantitative method and questionnaire survey. Although the research method is efficient and economical, it may affect the comprehensiveness of the research because the research method is relatively single. In the future, more qualitative researches are needed to further analyze the potential mechanism of college students’ learning stress, psychological resilience and learning burnout.

## Conclusions

In conclusion, we have found that college students have a medium level of learning pressure and learning burnout, and their level of psychological resilience is generally good. The learning pressure of college students is positively associated with the learning burnout of college students, learning burnout and learning stress are negatively correlated to the psychological resilience, psychological resilience may be very helpful to improve learning burnout and learning stress in college students. It is suggested that colleges and universities should take specific measures to relieve the learning pressure and create a good learning and living environment for them, so as to reduce the learning burnout of college students. Psychological resilience is the intermediary variable that learning pressure has a negative effect on learning burnout, that is, learning pressure plays a role in college students’ learning burnout through psychological resilience. Colleges mangers and heath care providers should take more practical methods to improve the psychological resilience of college students, which plays a very important role in alleviating the learning burnout of college students.

## Data Availability

All data generated or analyzed during this study are included in this published article.

## List of abbreviations

BMI: Body mass index
CFI: Comparative fit index
